# Phantom-based image quality assessment of clinical ^18^F-FDG protocols in digital PET/CT and comparison to conventional PMT-based PET/CT

**DOI:** 10.1186/s40658-019-0269-4

**Published:** 2020-01-06

**Authors:** Silvano Gnesin, Christine Kieffer, Konstantinos Zeimpekis, Jean-Pierre Papazyan, Renaud Guignard, John O. Prior, Francis R. Verdun, Thiago V. M. Lima

**Affiliations:** 1 Institute of Radiation physics, Lausanne University Hospital, University of Lausanne, Lausanne, Switzerland; 2 Department of nuclear medicine, Zürich Universitätsspital, Zurich, Switzerland; 3 Radiology and Medicine Nuclear Department, Genolier Clinique, Genolier, Switzerland; 4 Department of Nuclear Medicine, La Tour Medical Group, Meyrin, Switzerland; 5 Department of Nuclear Medicine and Molecular Imaging, Lausanne University Hospital, University of Lausanne, Bugnon 46, Lausanne, Switzerland; 6 Radiation Protection Group, Aarau Cantonal Hospital, Aarau, Switzerland

**Keywords:** Digital PET/CT, Image quality, Dose reduction, Protocol optimization

## Abstract

**Background:**

We assessed and compared image quality obtained with clinical ^18^F-FDG whole-body oncologic PET protocols used in three different, state-of-the-art digital PET/CT and two conventional PMT-based PET/CT devices.

Our goal was to evaluate an  improved trade-off between administered activity (patient dose exposure/signal-to-noise ratio) and acquisition time (patient comfort) while preserving diagnostic information achievable with the recently introduced digital detector technology compared to previous analogue PET technology.

**Methods:**

We performed list-mode (LM) PET acquisitions using a NEMA/IEC NU2 phantom, with activity concentrations of 5 kBq/mL and 25 kBq/mL for the background (9.5 L) and sphere inserts, respectively. For each device, reconstructions were obtained varying the image statistics (10, 30, 60, 90, 120, 180, and 300 s from LM data) and the number of iterations (range 1 to 10) in addition to the employed local clinical protocol setup. We measured for each reconstructed dataset: the quantitative cross-calibration, the image noise on the uniform background assessed by the coefficient of variation (COV), and the recovery coefficients (RCs) evaluated in the hot spheres. Additionally, we compared the characteristic time-activity-product (TAP) that is the product of scan time per bed position × mass-activity administered (in min·MBq/kg) across datasets.

**Results:**

Good system cross-calibration was obtained for all tested datasets with < 6% deviation from the expected value was observed. For all clinical protocol settings, image noise was compatible with clinical interpretation (COV < 15%). Digital PET showed an improved background signal-to-noise ratio as compared to conventional PMT-based PET. RCs were comparable between digital and PMT-based PET datasets. Compared to PMT-based PET, digital systems provided comparable image quality with lower TAP (from ~ 40% less and up to 70% less).

**Conclusions:**

This study compared the achievable clinical image quality in three state-of-the-art digital PET/CT devices (from different vendors) as well as in two conventional PMT-based PET. Reported results show that a comparable image quality is achievable with a TAP reduction of ~ 40% in digital PET. This could lead to a significant reduction of the administered mass-activity and/or scan time with direct benefits in terms of dose exposure and patient comfort.

## Background

Positron emission tomography (PET) coupled with computed tomography (CT) is an established quantitative imaging technique playing a key role in clinical oncology [1, 2]. In particular, quantitative or semi-quantitative ^18^F-FDG-PET/CT examinations cover a large part of PET indications, such as oncological, cardiac, and neurological imaging [3–5].

To guide clinical protocol validation and optimization, reference methodologies make use of phantoms with known geometry and activity preparation, representing a reasonable approximation of patient morphology and activity distribution [6]. To reproduce patient-relevant conditions, and to assess the signal recovery in small structures, the National Electrical Manufacturers Association (NEMA)/ International Electrotechnical Commission (IEC) NU2 phantom is currently a standard reference [7]. Phantoms with even more anthropomorphic shape also exist but they have not been widely tested so far and lack in standardization [8].

In the last decade, the clinical introduction of time of flight (TOF) technology and the point spread function (PSF) correction have substantially enhanced the achievable image quality [9–12].

In high-end commercial PET/CT devices, conventional analogue photomultipliers (PM) are replaced by the solid-state technology aiming to improve time resolution, event collection (consequently improving system sensitivity), localization, and counting efficiency [13].

In this evolving scenario, standardization and harmonization of ^18^F-FDG-PET protocols are essential to promote inter-machine and multi-center PET studies. Accordingly, image protocols have been proposed to satisfy the European Association of Nuclear Medicine (EANM)/Research 4 Life (EARL) recommendations [14, 15]. However, present EANM/EARL recommendations were derived for analogue PET systems and will undoubtedly be updated in the future to account for performances available in digital PET [16].

To the best of our knowledge, the image quality obtained with the three recently available commercial digital PET/CT devices using clinical whole-body oncologic ^18^F-FDG protocols have not been measured, characterized and compared yet in a single publication. Furthermore, the clinical image quality obtained with digital PET devices has not been extensively compared with analogue PET devices in a controlled and standardized approach.

Our aim was to present, characterize, and compare clinical implementation of ^18^F-FDG oncologic PET protocols across different PET technologies (digital vs. analogue). Accordingly, we performed NEMA/IEC NU2 phantom acquisitions on three recently installed digital TOF PET/CT systems (three different vendors) and compared the obtained results with the measurement performed in two analogue TOF PET/CT.

In addition, we also compared  the signal recovery obtained in hot sphere inserts of the NEMA/IEC NU2 phantom with present EAMN/EARL recommendations [17].

## Methods

### Phantom experiment design

The NEMA/IEC NU2 phantom (PTW, Freiburg, Germany) was used to characterize image quality and quantitative signal recovery in oncologic ^18^F-FDG-PET/CT images from three different digital PET/CT models: GE Healthcare Discovery-MI (GE Healthcare, Waukesha, USA) [13], Philips Vereos (Philips Medical Systems, Cleveland, USA) [18], and Siemens Biograph Vision 600 (Siemens Healthineers, Knoxville, USA) [19] and two analogue PET/CT devices: GE Healthcare Discovery 690 [20] and Siemens Biograph mCT [21].

The phantom's main volume (background) of 9.5 L mimics the human abdominal shape. It includes six spherical inserts with diameters of: 10, 13, 17, 22, 18, and 37 mm, respectively, and a lung insert (5-mm diameter and 16-cm long cylinder filled with plastic material mimiking the lung density of 0.3 g/mL) positioned in the center of the phantom to reproduce lung tissue attenuation.

The phantom was filled with a background activity concentration of 5 kBq/mL and an activity concentration five times higher (25 kBq/mL) in the spherical inserts. The background activity concentration reproduced the average hepatic activity concentration measured in patients occurring ^18^F-FDG oncologic PET 1 h after administration of a mass-activity of 3.5 MBq/kg, corresponding to the recommended dose reference level in Switzerland at the time of this study for this specific examination [22]. For each phantom experiment, on each tested PET/CT device, the net background activity concentration at the time of the image acquisition start was calculated from the net total activity injected in the known background volume.

### Clinical acquisition/reconstruction parameters

We performed step-and-shoot, single-bed, 300 s long list-mode (LM) PET acquisitions of the phantom in five PET centers in Switzerland. The phantom was placed on the PET bed with the equatorial plane of the spherical inserts at the center of the device field-of-view where the system sensitivity is expected to be maximal.

The LM data were reconstructed according to the local clinical protocol used for whole-body oncologic ^18^F-FDG PET examinations reported in Table 1.

**Table 1:** Systems, acquisition and reconstruction parameters applied in clinical whole-body oncologic ^18^F-FDG PET procedures

	Philips Vereos^d^	Siemens Vision^d^	Siemens mCT	GE Discovery-MI^d,a^	GE Discovery 690
System parameters					
Axial ring extent (mm)	164	261	221	250	153
Energy window (keV)	450–613	435–585	435–650	425–650	425–650
TOF’s resolution (ps)	316	215	540	370	544.3
NEMA System sensitivity (kcps/MBq)	5.6	16.4	9.6	22	7.5
Acquistion parameters					
Acq. Time (min)	1.5	2	2.5	2.5	1.5
Admin. Activity (MBq/kg)	2	2	5	1.5	3.5
Acq. Time (min) × A admin. (TAP in min × MBq/kg)	3	4	12.5	3.75	5.25
Reconstruction parameters					
Reconstruction methods	OSEM 3D TOF + PSF	OSEM 3D TOF + PSF	OSEM 3D TOF + PSF	OSEM 3D TOF + PSF	OSEM 3D TOF + PSF
Iterations and subsets (it,ss)	(3,15)/(2,10)	(4,5)	(3,21)	(3,16)	(3,16)
Filtre Gauss FWHM (mm)	0	0	3	6,4	5
Matrix size	144 × 144/288 × 288	440 × 440	512 × 512	256 × 256	256 × 256
Pixel size (mm)	4 × 4/2 × 2	1.65 × 1.65	1.59 × 1.59	2.73 × 2.73	2.73 × 2.73
Slice thickness (mm)	4/2	2	5	2.79	3.27

^a^In addition to OSEM, clinic FDG PET protocol for the GE Discovery MI also make use of the Q.Clear reconstruction algorithm (Q-param = 400)

^d^Digital PET systems

To investigate the influence of the image statistics, additional reconstructions were performed using time subsets of 10, 30, 60, 120, and 180 s obtained from the original 300 s long LM data.

Supplementary reconstructions were performed by varying the number of iterations from 1 to 10 to characterize the evolution of the signal recovery in background and spheres. Pertinent image corrections (normalization, dead time, activity decay, random coincidence, attenuation, and scatter corrections) were applied.

Some clinical reconstruction protocols do not use image smoothing. Therefore, to aid the comparison of image quality across tested devices, when applicable, image reconstruction without smoothing was also performed.

All devices used ordered subset expectation maximization (OSEM) based iterative reconstruction algorithm based on an iterations × subsets setup. Additionally, Discovery-MI’s data was also reconstructed with the Q.Clear algorithm [23] to correctly represent the local clinical practice. The Q.Clear reconstruction algorithm is a block sequential regularized EM algorithm with a single relaxation parameter and is not directly comparable with other algorithms in terms of the number of iterative updates.

In this study, we used the time (min) × mass activity (MBq/kg) product (TAP) as a metric for protocol characterization.

Table 1 also reports the TAP characteristic of each PET protocol tested. This parameter reflects the emission signal available for a given PET acquisition resulting from the product of the scan duration and the specific injected activity, two key parameters defining a clinical implementation of a PET procedure.

It is worth noting that different image matrices, different field of view (FOV) sizes, and therefore different pixel sizes were used across tested image protocols and PET devices.

### Background characterization

The PET-to-local dose calibrator cross-calibration (BG_cal_) was tested by calculating the ratio between the measured $\left({\overline{\boldsymbol{A}}}_{\boldsymbol{c},\boldsymbol{bg}}\right)$and expected average activity concentration (***A***_***c,bg***_) evaluated in the homogeneous phantom background:

$$ {\boldsymbol{BG}}_{\boldsymbol{c}\boldsymbol{al}}=\frac{{\overline{\boldsymbol{A}}}_{\boldsymbol{c},\boldsymbol{bg}}}{{\boldsymbol{A}}_{\boldsymbol{c},\boldsymbol{bg}}} $$

${\overline{\boldsymbol{A}}}_{\boldsymbol{c},\mathbf{bg}}$was the average activity concentration obtained by averaging the signal from the voxels contained in four cubic regions of interest (side of 40 mm) placed in the homogeneous background region surrounding the spheres. We consider as acceptable a deviation of < 0.1 from the ideal BG_cal_ = 1. The coefficient of variation (COV) used for image noise assessment was defined by the ratio between the standard deviation (SD_bg_) over all the voxels contained in the four cubic background VOIs and ${\overline{\boldsymbol{A}}}_{\boldsymbol{c},\mathbf{bg}}$:

$$ \boldsymbol{C}\mathbf{OV}\left(\%\right)=\frac{{\mathbf{SD}}_{\mathbf{bg}}}{{\overline{\mathbf{A}}}_{\mathbf{c},\mathbf{bg}}}\times \mathbf{100} $$

The background signal-to-noise ratio (SNR) is the reciprocal of the COV.

We considered a COV ≤ 15% (background SNR ≥ 6.7) as an acceptable noise level for clinical image interpretation as suggested in the EARL procedure [24]; even if this value is somehow arbitrary, it has already been used as a reference value in previously published works [14, 25, 26], which enables a term of comparison for ^18^F-FDG PET image quality assessments. COV as a function of TAP was assessed to investigate possible margins of optimization in terms of administered activity and/or scan time duration.

The COV for different values of TAP obtained by phantom experiments and TAP values for a COV = 15% were calculated by linear interpolation between neighboring measured values.

PET protocol setups were characterized by their specific TAP value. In particular, we reported and compared TAP obtained with clinical setup (TAP_clinic_) and TAP obtained for a matched image noise level by considering a COV = 15% (TAP_COV-15_).

### Spheres characterization

A cubic volume of interest (VOI), side of 50 mm, was centered on each spherical insert (*j =* 1,..,6) of the NEMA/IEC NU2 phantom. Maximum and background-adapted recovery coefficients (RC) were obtained as follows:

$$ {\mathrm{RC}}_{\mathrm{j},\max }=\frac{a_{\mathrm{c},\mathrm{sph},\mathrm{j},\max }}{{\mathrm{A}}_{\mathrm{c},\mathrm{sph}}} $$

$$ {\mathrm{RC}}_{\mathrm{j},\mathrm{A}50}=\frac{a_{\mathrm{c},\mathrm{sph},\mathrm{j},\mathrm{A}50}}{{\mathrm{A}}_{\mathrm{c},\mathrm{sph}}} $$

where *A*_*c*,sph_ is the expected activity concentration in the spheres, *a*_*c*,sph,*j*,max_ is the measured maximum voxel value (in Bq/mL) for a given spherical insert. *a*_*c*,sph,*j*,A50_ is the average voxel value in each hot insert VOI defined by a 3D iso-contour adapted for background as defined in [27] and recommended by the EANM Guidelines for FDG tumor PET imaging [28]. RCs were compared with reference values provided by the EANM/EARL accreditation protocol [17]. We tested the robustness RC_max_ and RC_A50_ as a function of time per bed position by comparing the measured values to the reference value obtained for the 300 s long acquisition.

Additional spherical VOIs, matching the actual insert volume, were segmented on the co-registered CT, to derive mean RCs:

$$ {\mathrm{RC}}_{j,\mathrm{mean}}=\frac{a_{c,\mathrm{sph},j,\mathrm{mean}}}{A_{c, sph}} $$

Convergence of signal recovery in spheres (*j* = 1,…,6) as a function of the number of iterative updates (UPD = iteration × subsets) was studied using the normalized value of RC_mean_:

$$ {\mathrm{RC}}_{j,\mathrm{mean},N}\left(\mathrm{UPD}\right)=\frac{{\mathrm{RC}}_{j,\mathrm{mean}}\left(\mathrm{UPD}\right)}{\max_{\mathrm{UPD}}\left({\mathrm{RC}}_{j,\mathrm{mean}}\right)} $$

where max_UPD_ (RC_j,mean_) is the maximum RC_mean_ value obtained for a given sphere (*j*) across the tested number of updates.

Image segmentation on PET data was performed using the PMOD (release 3.903) software (PMOD Technologies Ltd., Zurich, Switzerland).

Transaxial views across the equatorial plane of spherical inserts of the NEMA/IEC phantom, obtained for the tested clinical setups, are reported.

## Results

### Phantom experiment preparation

Parameters describing the experimental phantom preparation at the start of the PET acquisitions across the five tested PET devices are listed in Table 2.

**Table 2:** Average, standard deviation, and minimum and maximum values of activity concentrations present in the spherical inserts and main phantom background at the PET acquisition time start. The resulting sphere-to-background activity concentration ratio is also reported

	***A***_***c***,sph_ (kBq/mL)	***A***_***c***,bg_ (kBq/mL)	Sphere-to-bg ratio
Average (*n* = 5)	25.28	5.06	5.00
Standard deviation (SD)	1.25	0.27	0.27
Min	23.42	4.67	4.63
Max	26.45	5.29	5.46

### Background characterization

**Fig. 1:**
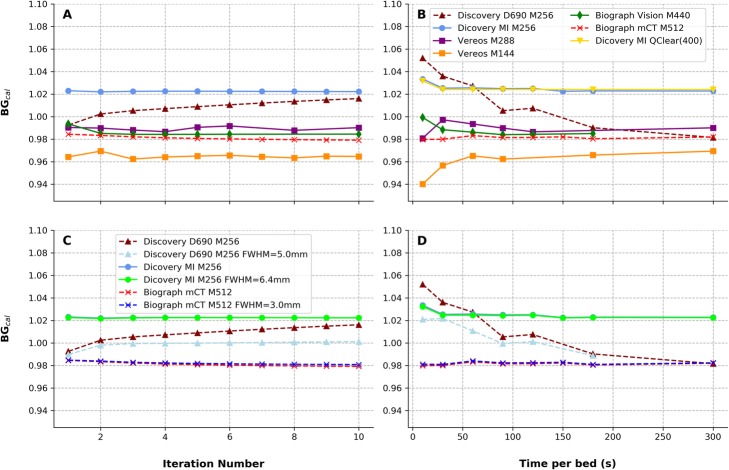
BG_cal_ as a function of the number of iterations used in the iterative reconstruction setups without Gaussian smoothing (**a**) and comparison of Gaussian vs. non-Gaussian setups (**c**) for devices that used the Gaussian smoothing in the clinic. Similarly, we reported BG_cal_ as a function of the time per bed position (**b** and **d**). Digital devices were labeled with full lines; dashed lines represent obtained results with analogue devices

The system cross-calibration (BG_cal_) as a function of the acquired statistics (by varying the time per bed position at matched total activity in the phantom) and the number of iterations used in the iterative reconstruction process, for the tested acquisition and reconstruction setups, is shown in Fig. 1.

The average BG_cal_ ± SD obtained across tested clinical protocol setups for the clinically used range of iterations (2 to 4 iterations) was BG_cal,2–4it_ = 0.992 ± 0.019 (range 0.963–1.023).

**Fig. 2:**
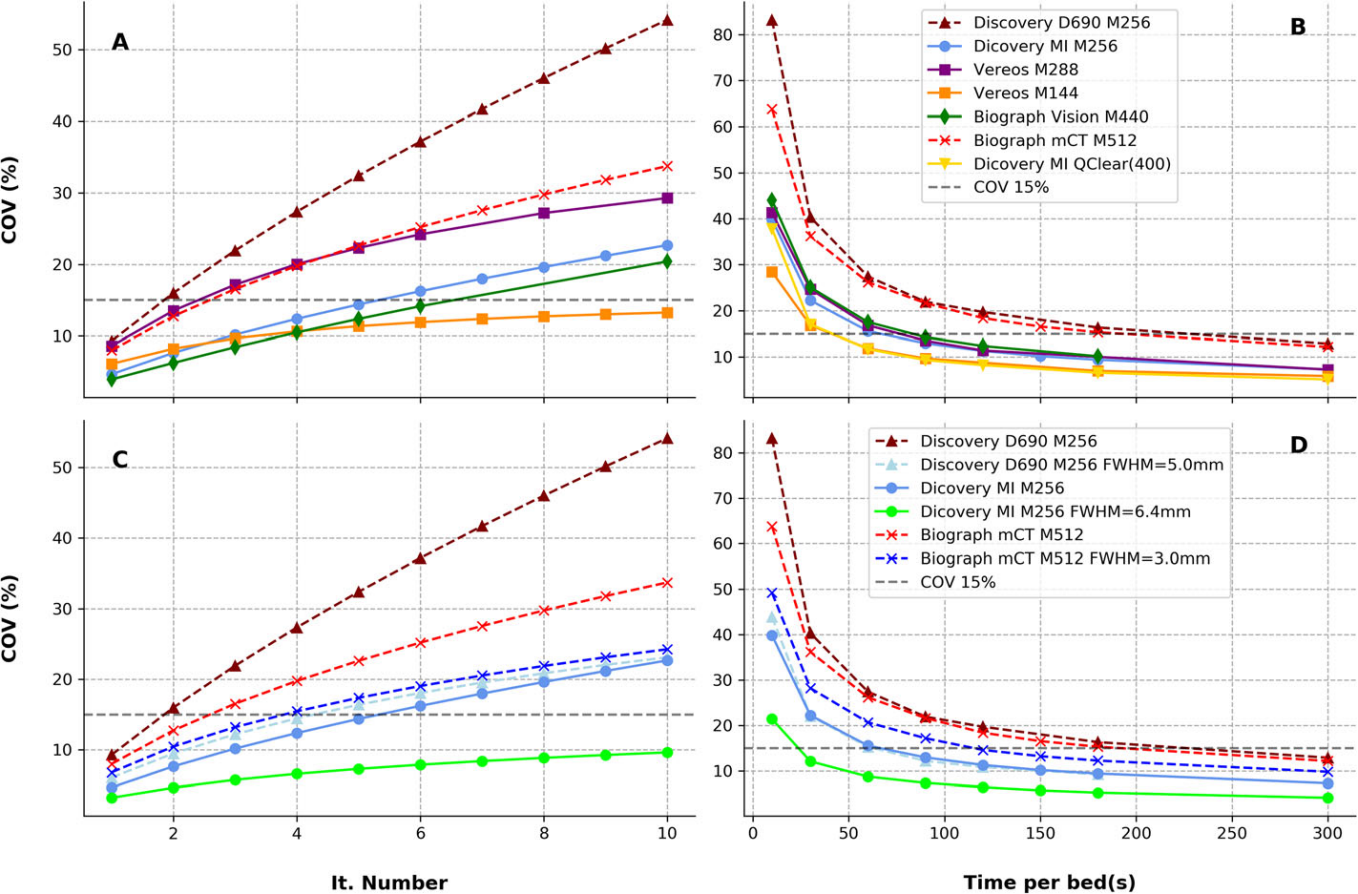
COV as a function of the number of iterations used in the iterative reconstruction setups without Gaussian smoothing (**a**) and comparison of Gaussian vs. non-Gaussian setups for devices that used the Gaussian smoothing in the clinic (**c**). Similarly, we reported the COV as a function of the time per bed position (**b** and **d**). Digital devices were labeled with full lines and dashed lines represent the obtained results with analogue devices

Measured COV values are reported in Fig. 2. The dashed black line indicates a 15% COV level (SNR = 6.7) used as an upper threshold defining an acceptable level of noise for clinical image interpretation. All tested clinical PET setups (Table 1) are characterized by a COV ≤ 15%.

**Fig. 3:**
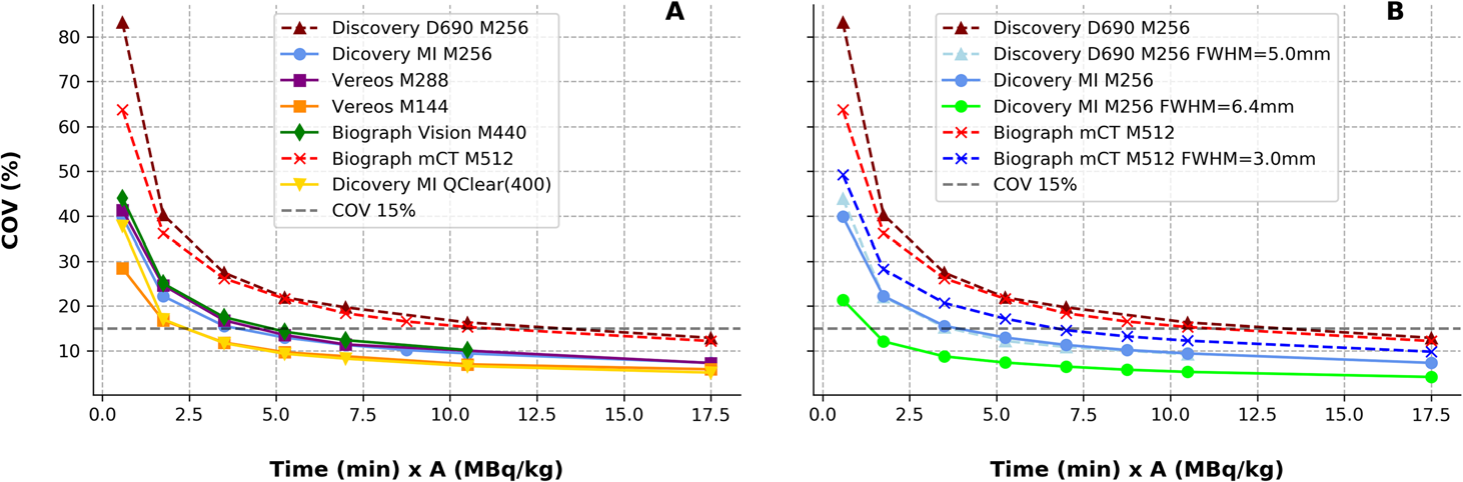
COV as a function of the time-activity-product (TAP) for all tested clinical setups. Iterative reconstruction setups without Gaussian smoothing (**a**) and comparison of Gaussian vs. non-Gaussian setups for devices that used the Gaussian smoothing in the clinic (**b**). Digital devices were labeled with full lines and dashed lines represent the obtained results with analogue devices

Figure 3 shows COV as a function of the TAP parameter. All clinical tested setups were characterized by a COV close to 15%. COV values corresponding to local clinical TAP and TAP values for a COV = 15% (TAP_COV-15_) are reported in Table 3.

**Table 3:** Clinical TAP (TAP_clinic_), COV values obtained for clinical TAP values, characteristic of tested ^18^F-FDG PET procedures, and TAP computed for a 15% COV level (TAP_COV-15_). Reconstruction protocol setups used in the clinic are labeled with (c)

PET device/recon. procedure	TAP_clinic_ (min × MBq/kg)	COV (%) at clinical TAP	TAP_COV-15_ (min × MBq/kg)
Discovery MI M256 FWHM = 6.4 mm (c)	3.75	8.6	1.4
Discovery MI M256 FWHM = 0 mm	3.75	15.2	3.9
Discovery Q.Clear (Q = 400) M256 (c)	3.75	11.4	2.4
Vereos M144 FWHM = 0 mm (c)	3	13.2	2.4
Vereos M288 FWHM = 0 mm (c)	3	19	4.5
Vision M440 FWHM = 0 mm (c)	4	14.1	3.5
Discovery 690 M256 FWHM = 5 mm (c)	5.25	12.2	3.7
Discovery 690 M256 FWHM = 0 mm	5.25	22	13.2
mCT M512 FWHM = 3 mm (c)	12.5	11.5	6.7
mCT M512 FWHM = 0 mm	12.5	14.4	11.2

Among the tested PET FDG protocols, two different image matrix sizes were used clinically with the Philips Vereos: 144 × 144 and 288 × 288, respectively. The TOF list-mode reconstruction [29, 30] leading to the thinner image discretization was characterized by a higher noise level: COV = 19% vs. 13.2% (clinical TAP of 3 min × MBq/kg). For a given device and same acquisition setups, lower COV levels were obtained using Gaussian image smoothing compared to not. Across clinical protocol setups, only the Vereos with the 288 × 288 image matrix had a clinical TAP lower than the TAP value corresponding to a 15% COV level (3 min × MBq/kg vs. 4.5 min × MBq/kg). The averaged TAP value corresponding to a 15% COV calculated across the clinical setups used in digital PET devices was 40% lower than the respective value calculated for the analogue PET (2.95 min × MBq/kg, range [1.4–4.5] vs. 5.2 min × MBq/kg, range [3.7–13.2]).

### Signal recovery in spheres

**Fig. 4:**
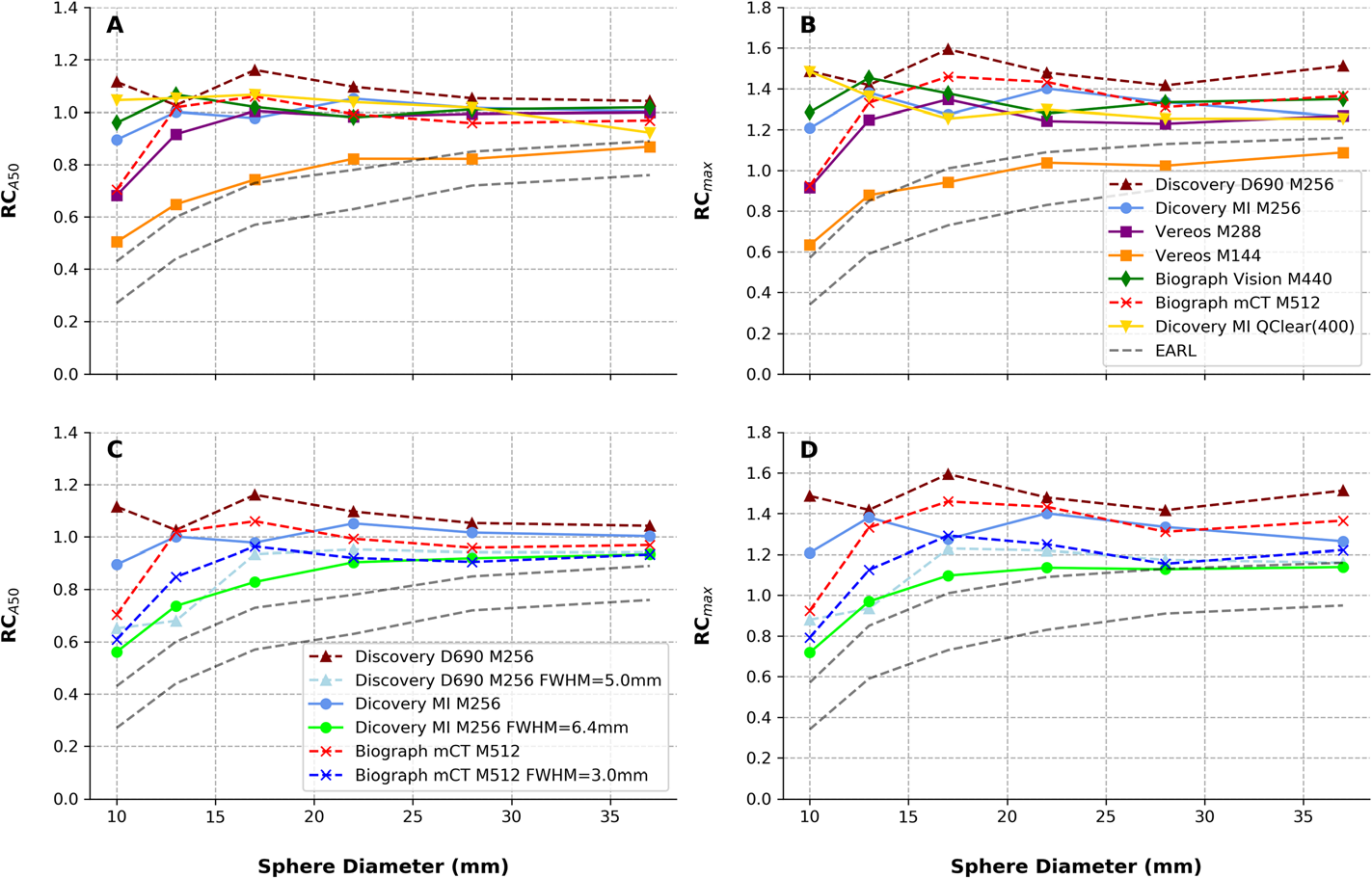
RC_A50_ and RC_max_ values as a function of the sphere diameter for acquisitions performed with a fixed scan duration of 180 s per bed position. Iterative reconstruction setups without Gaussian smoothing (**a** and **b**) and comparison of Gaussian vs. non-Gaussian setups for devices that used the Gaussian smoothing in the clinic (**c** and **d**). Digital devices were labeled with full lines and dashed lines represent the obtained results with analogue devices. Upper and lower RC boundaries specified by the EANM/EARL accreditation protocols are labeled with black dashed lines. EARL RC values (black dashed lines) refers to January 2017 version as reported in the EARL website [17]

Figure 4 shows RC_max_ and RC_A50_ values as a function of increasing sphere size for the PET setups tested using clinical reconstruction parameters (iterations × subsets and acquisition time) regardless of the image smoothing.

The convergence of the signal recovery in spheres of different sizes obtained as a function of the number of iterative updates is shown in Additional file 1: Figures S1 and S2.

As reported in Table 4, the normalized value of the RC_mean_ for a sphere of 10 mm (smaller size) and 17 mm (medium size) in diameter are respectively at least 89% and 95% of the maximum RC_mean_ values for the number of iterative updates used in clinical reconstruction setups. An improved convergence was measured for larger spheres.

**Table 4:** Normalized RC_mean_ for the number for iterative updates used in clinical reconstruction setups (and maximum RC_mean_ values) obtained for the smallest sphere insert (diameter of 10 mm) and a medium size insert (diameter of 17 mm) characteristic of tested PET FDG procedures. Reconstruction protocol setups used in the clinic are labeled with (c)

PET device/recon. procedure	Clinic setup, It × ss = UPD	RC_mean,***N***_ (max_UPD_(RC_mean_)), sphere 10 mm	RC_mean,***N***_ (max_UPD_(RC_mean_)), sphere 17 mm
Discovery MI M256 FWHM = 6.4 mm (c)	3 × 16 = 48	0.98 (0.48)	0.98 (0.74)
Discovery MI M256 FWHM = 0 mm	3 × 16 = 48	0.92 (0.65)	0.95 (0.87)
Vereos M144 FWHM = 0 mm (c)	3 × 15 = 45	0.98 (0.45)	1.0 (0.57)
Vereos M288 FWHM = 0 mm (c)	2 × 10 = 20	0.89 (0.61)	0.96 (0.78)
Vision M440 FWHM = 0 mm (c)	4 × 5 = 20	0.91 (0.60)	0.97 (0.84)
Discovery 690 M256 FWHM = 5 mm (c)	3 × 16 = 48	0.91 (0.54)	0.97 (0.78)
Discovery 690 M256 FWHM = 0 mm	3 × 16 = 48	0.89 (0.66)	0.94 (0.91)
mCT M512 FWHM = 3 mm (c)	3 × 21 = 63	0.92 (0.59)	0.98 (0.76)
mCT M512 FWHM = 0 mm	3 × 21 = 63	0.85 (0.64)	0.95 (0.80)

The robustness of RC_max_ and RC_A50_ according to the PET scan length was assessed for decreasing scan times (Additional file 1: Figure S2). Tested setups showed RCs to be stable (less than 15% variation compared to the reference value obtained for the 300-s bed acquisition scan time) for time per bed position ≥ 60 s.

**Fig. 5:**
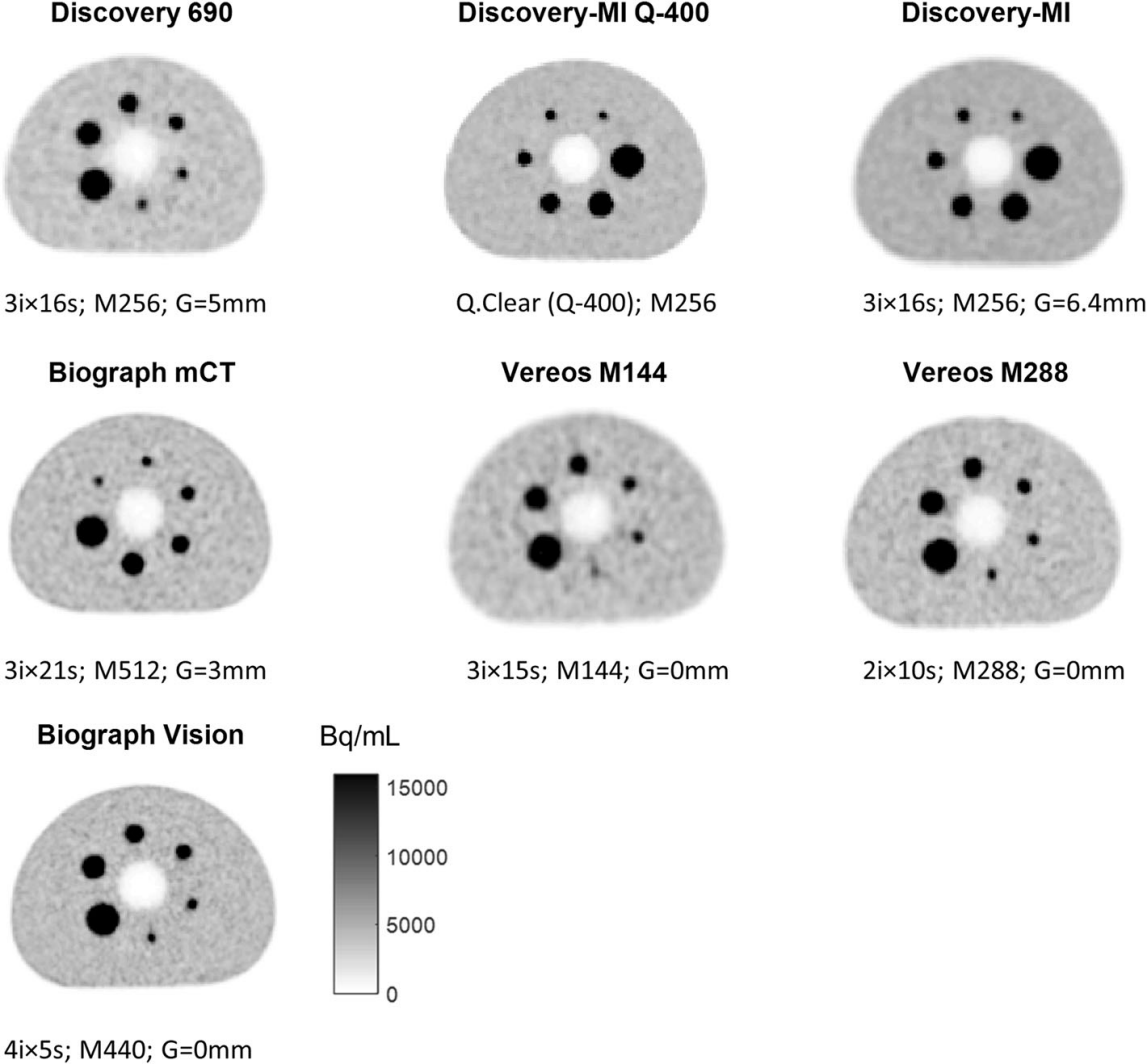
Transaxial views of the image quality of phantom images obtained with clinical setups

Transaxial views across the equatorial plane of the spherical inserts of the NEMA/IEC phantom, obtained for the tested clinical setups, are displayed in Fig. 5.

## Discussion

This study was the result of a collaboration among five PET centers in Switzerland. Data were collected from five different PET/CT devices: three recently installed (2017–2018) digital PET/CT and two analogue PET/CT installed in 2011 (GE Discovery 690) and 2010 (Siemens Biograph mCT), respectively. To the best of our knowledge, this is the first study comparing image quality from the three currently-available digital PET with those of the previous analogue generation. Although, absolute system performances have been compared elsewhere in the literature [19], the use of different acquisition and reconstruction parameters (ex. image matrix and pixel size, number of iterative updates), and the use of vendor-specific reconstruction algorithms make it difficult to disentangle the specific contribution of each parameters to the final image quality.

This study aimed to investigate and characterize the image quality of clinical whole-body oncologic ^18^F-FDG protocols. All tested setups included TOF information and PSF correction. The direct comparison of the detector technology was beyond the scope of this work.

Improved TOF capabilities and system sensitivity have been measured and reported in recent publications [13, 19, 31]. In particular, the gain in system sensitivity resulted from the interplay of the new digital technology coupled with the adoption of an improved axial extension of the PET detector by some of the available models.

We based our study on PET acquisitions and reconstruction of a NEMA/IEC NU2 body phantom, which is a standard in PET image quality assessments. The phantom was prepared with a good reproducibility across centers as reported in Table 2. PET datasets were obtained by varying the number of iterations to verify signal recovery convergence and the scan acquisition time to verify image quality stability as a function of collected statistics. To remove the influence of PVE effect due to image smoothing, we produced PET data without post-reconstruction smoothing when the local reconstruction setup was adopting it.

All tested devices and reconstruction setups demonstrate a good cross-calibration with the local dose calibrator. Deviations from BG_cal_ = 1 were always less than 6% regardless of the time per bed position (10–300 s) and the number of iterations (1 to 10). Quantitative bias increased at low count density (as visible in Fig. 1); this behavior was already documented and characterized in the literature [32–34]. Furthermore, the bias observed at low count density was found to have a trend for lower levels when a list-mode based reconstruction was used (Vereos system) while this trend was to higher values when the reconstruction methods were based on sinograms. This behavior was also described in the literature in conditions of low count statistics such as ^90^Y PET [35], PET for ion-beam therapy monitoring [36], and low-dose ^18^F-FDG PET [37].

As expected, image noise increased with the number of iterative reconstruction updates (Fig. 2a, c). In the tested conditions, digital PET systems exhibited a lower noise level compared to analogue PET systems. This was more evident when comparing reconstruction setups without Gaussian filtering (Fig. 2c, d). This feature may be potentially attributed to the synergistic improved system sensitivity and TOF performances of digital devices compared to analogue PET. Image noise as a function of the acquired statistics (Fig. 2b, d), also confirmed the superior noise properties of digital PET images vs. analogue devices. In particular, we used a 15% COV level as a reference maximum noise level for clinical evaluation as suggested in the literature [14, 25, 27]. We found all tested clinical protocol setups characterized by a COV ≤ 15% for the adopted experimental setup representing a mass-activity administration of 3.5 MBq/kg.

We reported the COV as a function of the TAP (Fig. 3). TAP values characteristic of local clinical image protocols (TAP_clinic_, summarized in Tables 1 and 3) resulted in COV close to 15% (range 9–19%). Based on this result, we can deduce that the tested setups satisfy the requirements for clinical interpretation. Nevertheless, the assumed reference limit, COV = 15%, is somehow arbitrary; therefore, the particular image pattern, signal recovery in lesions, and different clinical experience between sites and devices would motivate possibly different optimal COV values for clinical image evaluation. It is also worth remarking that COV alone does not represent the most significant metric for comparing image quality across devices and protocol setups, since this parameter depends not only on overall device performance and reconstruction parameters but also on the injected specific mass-activity and the adopted scan time duration per bed position. For this reason, we adopted the TAP_COV-15_ as a term of comparison between different technologies. The COV obtained at clinical TAP, however, was reported to characterize the different clinical protocols.

Our results confirm lower TAP_clinic_ (range 3–4 min·MBq/kg) and are currently used with digital PET devices compared to the tested analogue PET (TAP_clinic_ = 5.25 min × MBq/kg or higher). On average, a 40% TAP reduction was reported in clinical configurations in favor of digital PET.

When considering TAP_COV-15_, if we exclude the mCT device (thus considering it an outlier), analogue systems are represented by the only value of 3.7 min × MBq/kg used with the Discovery 690 (M256, Gaussian smoothing FWHM = 5 mm). Accordingly, the average TAP_COV-15_ obtained from digital PET systems (2.84 min × MBq/kg, range 1.4–4.5 min × MBq/kg) is 23% lower than the TAP_COV-15_ obtained for the analogue Discovery 690.

We should also consider that the clinical setup adopted for the Discovery 690 includes a Gaussian smoothing (FWHM = 5 mm) that helps reducing TAP_COV-15_ values, while, excluding the Discovery MI (M256, FWHM = 6.4 mm) setup, all other clinical setups adopted in digital devices did not used Gaussian smoothing.

The differential improvement of new systems is even more evident when comparing similar setups without the use of Gaussian smoothing. For instance, according to data reported in Table 3, comparing the Discovery 690 with the Discovery-MI that used the same image matrix (256 × 256) and iterations × subsets (3 × 16), the TAP_COV-15_was 13.2 min × MBq/kg and 3.9 min × MBq/kg, respectively, corresponding to a 70% TAP_COV-15_ reduction in favor of the digital PET system. This translates to a lower mass-activity administration and/or shorter scan times at matched image noise levels. Accordingly, patient comfort (at matched image quality) can be improved and/or dose exposure reduction can be achieved as discussed in the recent clinic works of Behr et al. [38] and Van Sluis et al. [39].

RC_max_ and RC_A50_ (Fig. 4) higher than the present reference EANM/EARL levels were commonly obtained in all clinical protocol setups tested. These values are typical of PET reconstructions adopting TOF and PSF corrections [26].

The EARL proposed a target range of RCs to promote inter-device and inter-center comparison of quantitative PET data. This is not always the purpose in local clinical setups. Most often, the local clinical demand favor image contrast and spatial resolution (reduced PVE) with resulting higher RCs values compared to the proposed EARL range.

Lower RC levels were observed for the clinical setups in the Discovery MI, as a consequence of the 6.4-mm Gaussian filter applied and for the Vereos system adopting the 144 × 144 matrix size which results in large voxels with consequent large PVE in small structures. By definition, RC_max_ and RC_A50_ depend on the voxel with the maximum value and are intrinsically sensitive to the image noise level. Accordingly, we observed they increased with the number of iterations and decreased image statistics (Additional file 1: Figures S3 and S4) especially for reconstruction protocols without image smoothing. For the tested conditions, an important deviation of RC_max_ and RC_A50_ (higher RCs) can arise for scan times shorter than 60 s.

A normalized RC_mean_ was used to test the signal recovery convergence as a function of the number of iterative updates. Across the tested clinic protocol setups, a reasonable level of convergence (RC_mean,*N*_ ≥ 89%) was obtained even for the smallest spherical insert (10 mm in diameter). In particular, there are two systems exhibiting a faster convergence rate: the Biograph Vision and the Vereos, the first probably due to the best TOF timing resolution (214 ps), and the latter probably thanks to the favorable convergence properties of the blob-based OSEM iterative reconstruction algorithm [40], having a TOF time resolution of 316 ps, an intermediate value compared to the Siemens and the GE digital systems. We also observed faster convergence for reconstruction setups adopting Gaussian smoothing compared to reconstruction without smoothing. This behavior can be attributed to the peculiarity of image smoothing in reducing high spatial frequency (typical of small structures) that are known to require more iterations to converge when compared to lower spatial frequency (characterizing large structures) that is also the reasons why this behavior is more evident for the spheres of smaller size.

Compared to the tested OSEM iterative reconstruction setups, the Q.Clear implemented in the Discovery-MI PET/CT showed (at least) comparable performances. This reconstruction method indeed, guarantees a good level of signal recovery coupled with favorable noise properties. It was not our goal in this work to systematically characterize the Q.Clear algorithm, something which has been discussed elsewhere in the literature [24, 40].

Concerning the signal recovery performances, we did not observe major differences between conventional PMT-based PET and recently introduced digital PET devices (all reconstructions used PSF correction). The work of Kaalep et al. [26] pointed out the convenience of adopting a new range of signal recovery coefficients that thanks to the inclusion of PSF all PET devices can achieve. Kaalep et al. tested analogue PET devices, but in light of the results presented in our work, their methodology and results are in principle transferable to recently available digital PET.

We also noticed that, thanks to the improved system sensitivity and TOF capabilities, clinical protocols implemented in digital PET devices tend to avoid image smoothing. This fact coupled with the use of a relatively small voxel size (ex. 1.65 × 1.65 × 2 mm^3^ for the tested Biograph Vision device) can help reducing partial volume effects. Consequently, based on our results, we expect at a matched activity distribution present across the device FOV, and at a matched acquisition time duration, the digital PET potentially provide higher contrast-to-noise ratios, thus possibly improving lesions detection and quantitative accuracy.

## Limitations

Despite the limited number of tested PET devices, digital PET (*n* = 3) and analogue PET (*n* = 2), we found reasonable indications on the potential of operating digital devices at lower TAP compared to conventional analogue ones at matched image quality. Furthermore, matched image quality was achievable (for instance COV = 15%, as used in our study) in digital PET without applying additional image smoothing and/or using smaller voxel size with potential benefit in reducing PVE.

## Conclusion

This work is the result of the collaboration of different PET centers in Switzerland and was, to the best of our knowledge, the first study comparing the image quality obtained for clinical whole-body oncologic ^18^F-FDG PET protocols using the three recently introduced digital PET devices. We further extended the comparison to two analogue PET devices equipped with conventional PMTs. The methodology, based on a well-characterized NEMA/IEC NU2 phantom, highlighted the improved signal-to-noise ratios achievable with the new digital PET devices compared to conventional ones. With appropriate protocol optimization in terms of acquisition and reconstruction parameters, we found that sensible improvements in patient comfort (reduced scan time for the same matched image quality) and/or dose exposure (reduced administered activity) are achievable.

## Supplementary information

**Additional file 1: Figure S1.** Signal recovery convergence in spheres of different size for iterative reconstruction setups without Gaussian smoothing. Reconstruction setups implemented in digital devices were labeled with full lines, dashed lines denotes reconstruction setups implemented in analogue devices. **Figure S2** Signal recovery convergence in spheres of different size. Comparison of Gaussian vs. non-Gaussian setups for devices that used the Gaussian smoothing in clinic. Reconstruction setups implemented in digital devices were labeled with full lines, dashed lines denotes reconstruction setups implemented in analogue devices. **Figure S3.** RC_max_ as function of acquisition time for the different spheres, for iterative reconstruction setups without Gaussian smoothing Reconstruction setups implemented in digital devices were labeled with full lines, dashed lines denotes reconstruction setups implemented in analogue devices. Upper and lower RC boundaries specified by the EANM/EARL accreditation protocols are labelled with black dashed lines. EARL RC values (black dashed lines) refers to January 2017 version as reported in the EARL website [17]. **Figure S4.** RC_max_ as function of acquisition time for the different spheres. Comparison of Gaussian vs. non-Gaussian setups for devices that used the Gaussian smoothing in clinic. Reconstruction setups implemented in digital devices were labeled with full lines, dashed lines denotes reconstruction setups implemented in analogue devices. Upper and lower RC boundaries specified by the EANM/EARL accreditation protocols are labelled with black dashed lines. EARL RC values (black dashed lines) refers to January 2017 version as reported in the EARL website [17].

## Data Availability

The datasets used and/or analyzed during the current study are available from the corresponding author on reasonable request.

## Abbreviations

COV: Coefficient of variation
CT: Computed tomography
EANM: European Association of Nuclear Medicine
EARL: Research 4 Life
FDG: Fluorodeoxyglucose
FOV: Field of view
IEC: International Electrotechnical Commission
LM: List-mode
NEMA: National Electrical Manufacturers Association
OSEM: Ordered subset expectation maximization
PET: Positron emission tomography
PM: Photo multiplier
PMT: Photo multiplier tube
PSF: Point spread function
RC: Recovery coefficient
SD: Standard deviation
SNR: Signal-to-noise ratio
TAP: Time-activity-product
TOF: Time of flight
UPD: Number of iterative updates
VOI: Volume of interest

## References

[1] Czernin J, Allen-Auerbach M, Nathanson D, Herrmann K (2013). PET/CT in oncology: current status and perspectives. Curr Radiol Rep.

[2] Farwell MD, Pryma DA, Mankoff DA (2014). PET/CT imaging in cancer: current applications and future directions. Cancer.

[3] Zhuang H, Codreanu I (2015). Growing applications of FDG PET-CT imaging in non-oncologic conditions. J Biomed Res.

[4] Skali H, Schulman AR, Dorbala S (2013). 18F-FDG PET/CT for the assessment of myocardial sarcoidosis. Curr Cardiol Rep.

[5] Herholz K (2014). The role of PET quantification in neurological imaging: FDG and amyloid imaging in dementia. Clin Transl Imaging.

[6] AGENCY IAE. Quality assurance for PET and PET/CT systems. 2009;Human Health Series No. 1, IAEA, Vienna (2009).(Human Health Series No. 1, IAEA, Vienna (2009).

[7] National Electrical Manufacturers Association Rosslyn VA. NEMA Standards Publication NU 2-2012, Performance measurements of positron emission tomographs. 2012.

[8] Gear JI, Cummings C, Craig AJ, Divoli A, Long CD, Tapner M (2016). Abdo-Man: a 3D-printed anthropomorphic phantom for validating quantitative SIRT. EJNMMI Phys.

[9] Akamatsu G, Ishikawa K, Mitsumoto K, Taniguchi T, Ohya N, Baba S (2012). Improvement in PET/CT image quality with a combination of point-spread function and time-of-flight in relation to reconstruction parameters. J Nucl Med.

[10] Taniguchi T, Akamatsu G, Kasahara Y, Mitsumoto K, Baba S, Tsutsui Y (2015). Improvement in PET/CT image quality in overweight patients with PSF and TOF. Ann Nucl Med.

[11] Vandenberghe S, Mikhaylova E, D'Hoe E, Mollet P, Karp JS (2016). Recent developments in time-of-flight PET. EJNMMI Phys.

[12] Karp JS, Surti S, Daube-Witherspoon ME, Muehllehner G (2008). Benefit of time-of-flight in PET: experimental and clinical results. J Nucl Med.

[13] Hsu DFC, Ilan E, Peterson WT, Uribe J, Lubberink M, Levin CS (2017). Studies of a next-generation silicon-photomultiplier-based time-of-flight PET/CT system. J Nucl Med.

[14] Graham MM, Wahl RL, Hoffman JM, Yap JT, Sunderland JJ, Boellaard R (2015). Summary of the UPICT protocol for 18F-FDG PET/CT imaging in oncology clinical trials. J Nucl Med.

[15] Koopman D, van Osch JA, Jager PL, Tenbergen CJ, Knollema S, Slump CH (2016). Technical note: how to determine the FDG activity for tumour PET imaging that satisfies European guidelines. EJNMMI Phys.

[16] van der Vos CS, Koopman D, Rijnsdorp S, Arends AJ, Boellaard R, van Dalen JA (2017). Quantification, improvement, and harmonization of small lesion detection with state-of-the-art PET. Eur J Nucl Med Mol Imaging.

[17] website. EE. Available from: http://earl.eanm.org/cms/website.php?id=/en/projects/fdg_pet_ct_accreditation/accreditation_specifications.htm

[18] Rausch I, Ruiz A, Valverde-Pascual I, Cal-Gonzalez J, Beyer T, Carrio I (2019). Performance evaluation of the Vereos PET/CT system according to the NEMA NU2-2012 standard. J Nucl Med..

[19] van Sluis JJ, de Jong J, Schaar J, Noordzij W, van Snick P, Dierckx R, et al. Performance characteristics of the digital Biograph Vision PET/CT system. J Nucl Med. 2019. 10.2967/jnumed.118.215418 30630944

[20] Bettinardi V, Presotto L, Rapisarda E, Picchio M, Gianolli L, Gilardi MC (2011). Physical performance of the new hybrid PETCT Discovery-690. Med Phys..

[21] Jakoby BW, Bercier Y, Conti M, Casey ME, Bendriem B, Townsend DW (2011). Physical and clinical performance of the mCT time-of-flight PET/CT scanner. Phys Med Biol..

[22] OFSP Ofdlsp. Directive L-08-01 Niveaux de référence diagnostiques (NRD) fixés pour les examens de médecine nucléaire. Available from: https://www.bag.admin.ch/dam/bag/fr/dokumente/str/fanm/weisungen-merkblaetter/in-kraft/l-08-01.pdf.download.pdf/L-08-01_FR.pdf.

[23] Sah BR, Stolzmann P, Delso G, Wollenweber SD, Hullner M, Hakami YA (2017). Clinical evaluation of a block sequential regularized expectation maximization reconstruction algorithm in 18F-FDG PET/CT studies. Nucl Med Commun..

[24] Boellaard R, Willemsen AT, Arends B, Visser EP. EARL procedure for assessing PET/CT system specific patient FDG activity preparations for quantitative FDG PET/CT studies. 2013:1-3.

[25] Kaalep A, Sera T, Rijnsdorp S, Yaqub M, Talsma A, Lodge MA (2018). Feasibility of state of the art PET/CT systems performance harmonisation. Eur J Nucl Med Mol Imaging.

[26] Boellaard R, Delgado-Bolton R, Oyen WJ, Giammarile F, Tatsch K, Eschner W (2015). FDG PET/CT: EANM procedure guidelines for tumour imaging: version 2.0. Eur J Nucl Med Mol Imaging.

[27] Boellaard R, Krak NC, Hoekstra OS, Lammertsma AA (2004). Effects of noise, image resolution, and ROI definition on the accuracy of standard uptake values: a simulation study. J Nucl Med.

[28] Boellaard R, O'Doherty MJ, Weber WA, Mottaghy FM, Lonsdale MN, Stroobants SG (2010). FDG PET and PET/CT: EANM procedure guidelines for tumour PET imaging: version 1.0. Eur J Nucl Med Mol Imaging.

[29] Wang W, Hu Z, Gualtieri EE, Parma MJ, Walsh ES, Sebok D, et al. Systematic and distributed time-of-flight list mode PET reconstruction. Ieee Nucl Sci Conf R. 2006:1715–22.

[30] Kadrmas DJ (2004). LOR-OSEM: statistical PET reconstruction from raw line-of-response histograms. Phys Med Biol.

[31] Zhang J, Maniawski P, Knopp MV (2018). Performance evaluation of the next generation solid-state digital photon counting PET/CT system. EJNMMI Res..

[32] Jian Y, Planeta B, Carson RE (2015). Evaluation of bias and variance in low-count OSEM list mode reconstruction. Phys Med Biol.

[33] van Velden FH, Kloet RW, van Berckel BN, Lammertsma AA, Boellaard R (2009). Accuracy of 3-dimensional reconstruction algorithms for the high-resolution research tomograph. J Nucl Med.

[34] Carlier T, Willowson KP, Fourkal E, Bailey DL, Doss M, Conti M (2015). (90) Y -PET imaging: Exploring limitations and accuracy under conditions of low counts and high random fraction. Med Phys.

[35] Kurz C, Bauer J, Conti M, Guerin L, Eriksson L, Parodi K (2015). Investigating the limits of PET/CT imaging at very low true count rates and high random fractions in ion-beam therapy monitoring. Med Phys.

[36] Schaefferkoetter JD, Yan J, Sjoholm T, Townsend DW, Conti M, Tam JK (2017). Quantitative accuracy and lesion detectability of low-dose (18)F-FDG PET for lung cancer screening. J Nucl Med.

[37] Behr SC, Bahroos E, Hawkins RA, Nardo L, Ravanfar V, Capbarat EV (2018). Quantitative and visual assessments toward potential sub-mSv or ultrafast FDG PET using high-sensitivity TOF PET in PET/MRI. Mol Imaging Biol.

[38] van Sluis J, Boellaard R, Dierckx RA, Stormezand G, Glaudemans A, Noordzij W. Image quality and activity optimization in oncological (18)F-FDG PET using the digital Biograph Vision PET/CT. J Nucl Med. 2019. 10.2967/jnumed.119.234351 31628214

[39] Teoh EJ, McGowan DR, Macpherson RE, Bradley KM, Gleeson FV (2015). Phantom and clinical evaluation of the Bayesian penalized likelihood reconstruction algorithm Q.Clear on an LYSO PET/CT system. J Nucl Med.

[40] Walker MD, Asselin MC, Julyan PJ, Feldmann M, Talbot PS, Jones T (2011). Bias in iterative reconstruction of low-statistics PET data: benefits of a resolution model. Phys Med Biol..

