# Supplementary material for: Phantom-based image quality assessment of clinical 18F-FDG protocols in digital PET/CT and comparison to conventional PMT-based PET/CT
Source: EJNMMI Phys. 2020 Jan 6;7:1. doi: 10.1186/s40658-019-0269-4 (PMC6944719; doi:10.1186/s40658-019-0269-4)

Supplemental data

Supplemental Figure S1 Signal recovery convergence in spheres of different size for iterative reconstruction setups without Gaussian smoothing. Reconstruction setups implemented in digital devices were labeled with full lines, dashed lines denotes reconstruction setups implemented in analogue devices.


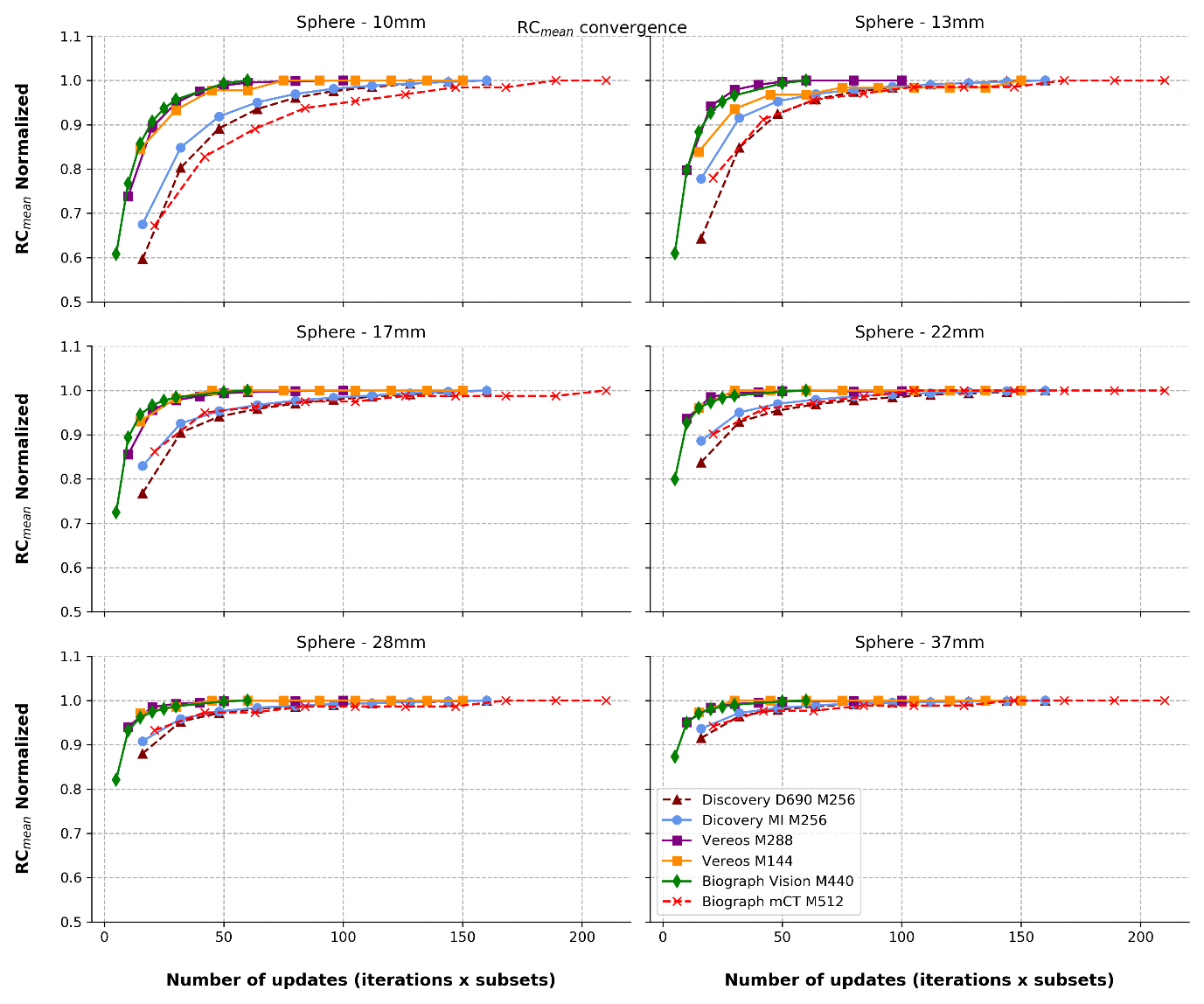


Supplemental Figure S2 Signal recovery convergence in spheres of different size. Comparison of Gaussian vs. non-Gaussian setups for devices that used the Gaussian smoothing in clinic. Reconstruction setups implemented in digital devices were labeled with full lines, dashed lines denotes reconstruction setups implemented in analogue devices.


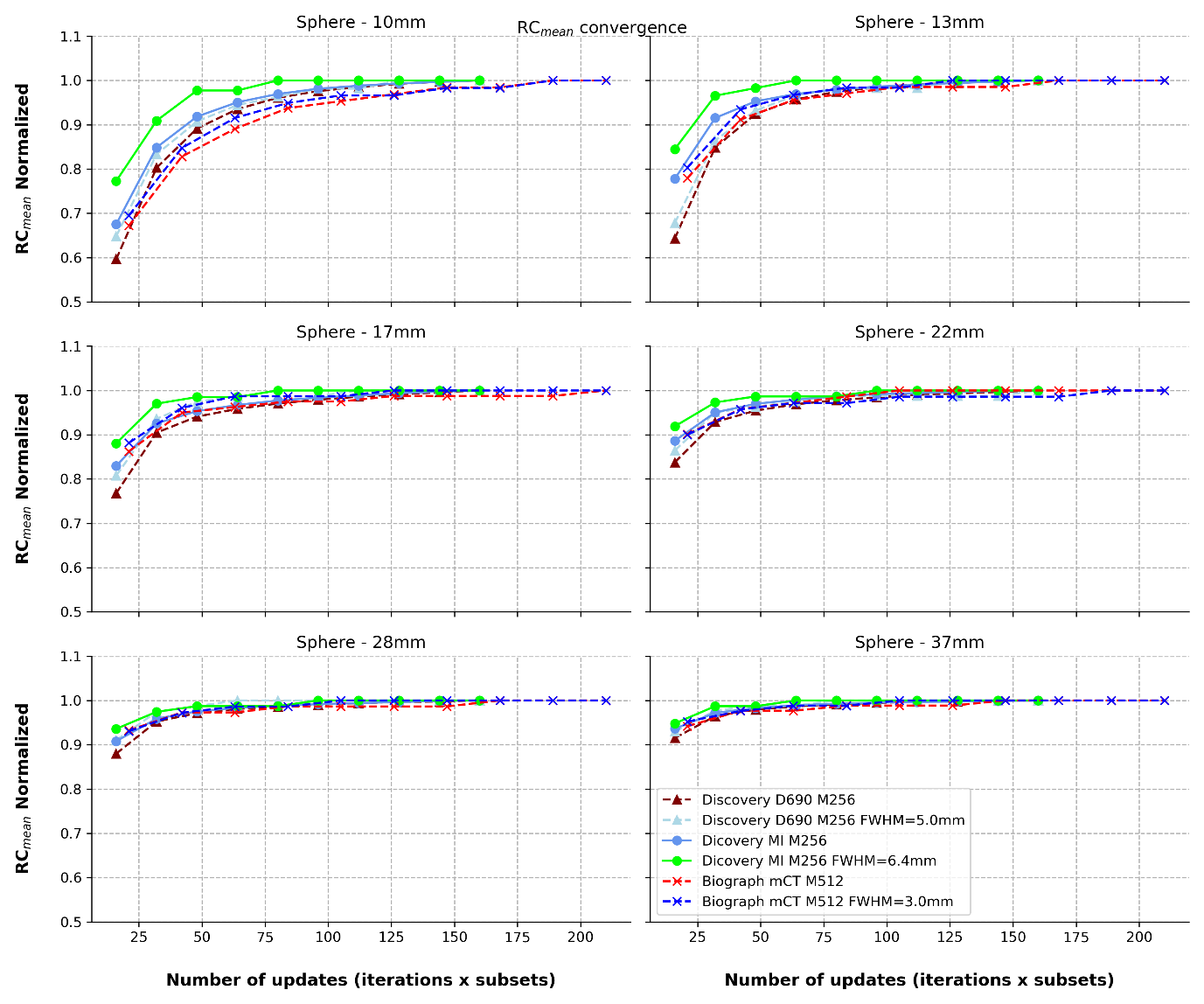


Supplemental Figure S3. RC_max_ as function of acquisition time for the different spheres, for iterative reconstruction setups without Gaussian smoothing Reconstruction setups implemented in digital devices were labeled with full lines, dashed lines denotes reconstruction setups implemented in analogue devices. Upper and lower RC boundaries specified by the EANM/EARL accreditation protocols are labelled with black dashed lines. EARL RC values (black dashed lines) refers to January 2017 version as reported in the EARL website (17).


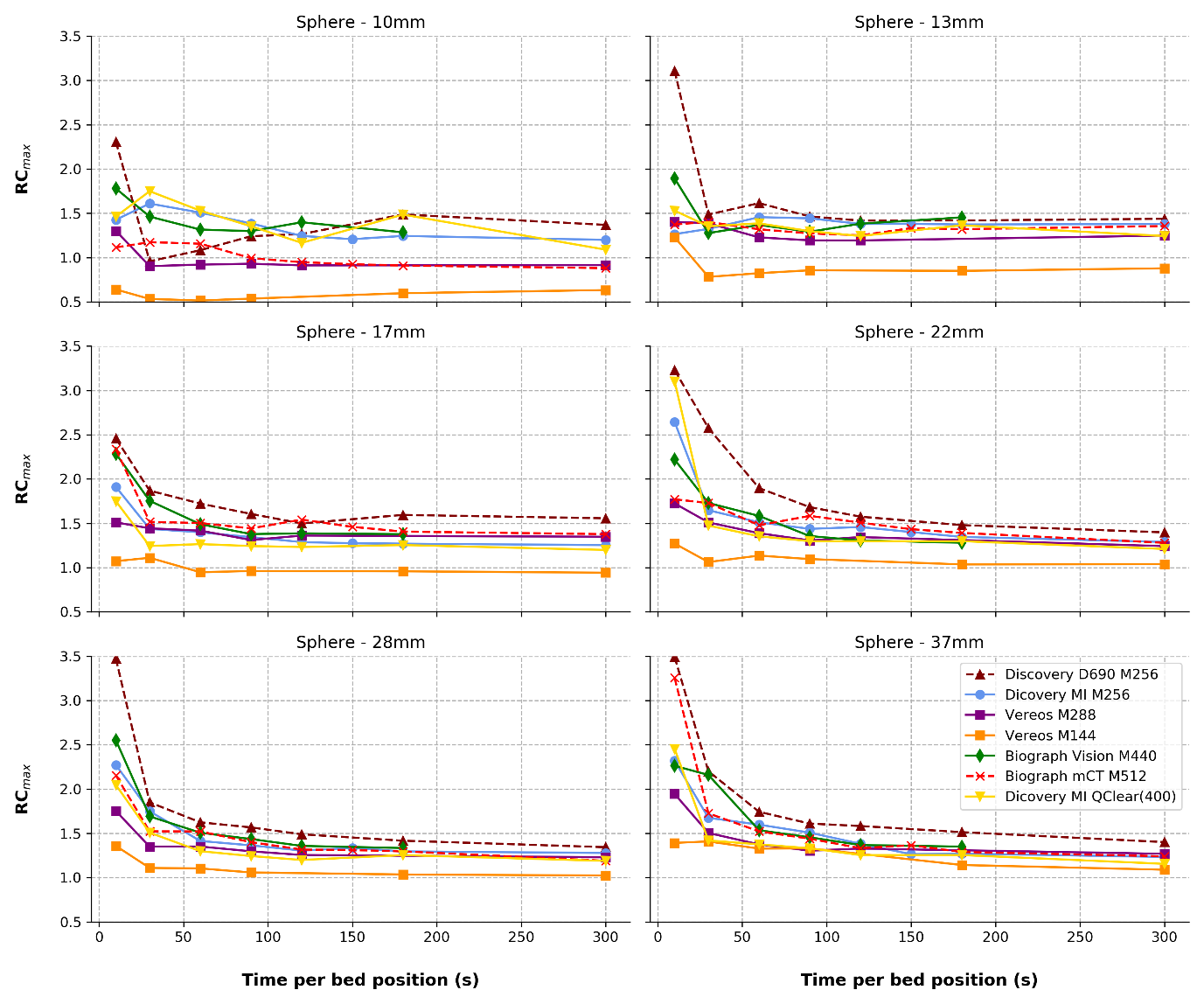


Supplemental Figure S4. RC_max_ as function of acquisition time for the different spheres. Comparison of Gaussian vs. non-Gaussian setups for devices that used the Gaussian smoothing in clinic. Reconstruction setups implemented in digital devices were labeled with full lines, dashed lines denotes reconstruction setups implemented in analogue devices. Upper and lower RC boundaries specified by the EANM/EARL accreditation protocols are labelled with black dashed lines. EARL RC values (black dashed lines) refers to January 2017 version as reported in the EARL website (17).


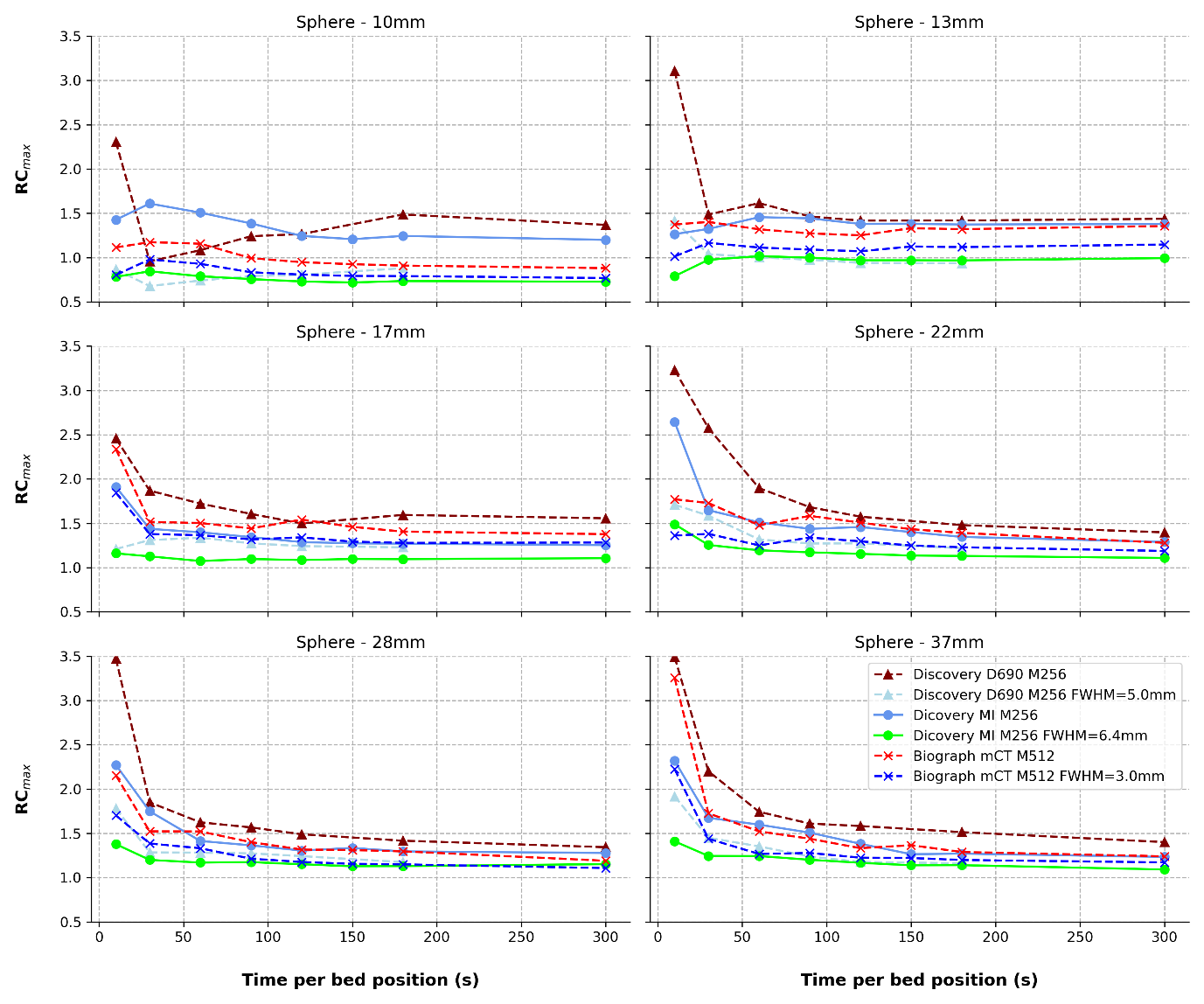

Supplement: Supplementary file 1 — Additional file 1: Figure S1. Signal recovery convergence in spheres of different size for iterative reconstruction setups without Gaussian smoothing. Reconstruction setups implemented in digital devices were labeled with full lines, dashed lines denotes reconstruction setups implemented in analogue devices. Figure S2 Signal recovery convergence in spheres of different size. Comparison of Gaussian vs. non-Gaussian setups for devices that used the Gaussian smoothing in clinic. Reconstruction setups implemented in digital devices were labeled with full lines, dashed lines denotes reconstruction setups implemented in analogue devices. Figure S3. RCmax as function of acquisition time for the different spheres, for iterative reconstruction setups without Gaussian smoothing Reconstruction setups implemented in digital devices were labeled with full lines, dashed lines denotes reconstruction setups implemented in analogue devices. Upper and lower RC boundaries specified by the EANM/EARL accreditation protocols are labelled with black dashed lines. EARL RC values (black dashed lines) refers to January 2017 version as reported in the EARL website [17]. Figure S4. RCmax as function of acquisition time for the different spheres. Comparison of Gaussian vs. non-Gaussian setups for devices that used the Gaussian smoothing in clinic. Reconstruction setups implemented in digital devices were labeled with full lines, dashed lines denotes reconstruction setups implemented in analogue devices. Upper and lower RC boundaries specified by the EANM/EARL accreditation protocols are labelled with black dashed lines. EARL RC values (black dashed lines) refers to January 2017 version as reported in the EARL website [17]. [file 40658_2019_269_MOESM1_ESM.docx]
